# Pilot study of a cluster randomised trial of a guided e-learning health promotion intervention for managers based on management standards for the improvement of employee well-being and reduction of sickness absence: GEM Study

**DOI:** 10.1136/bmjopen-2015-007981

**Published:** 2015-10-26

**Authors:** Stephen A Stansfeld, Sally Kerry, Tarani Chandola, Jill Russell, Lee Berney, Natalia Hounsome, Doris Lanz, Céire Costelloe, Melanie Smuk, Kamaldeep Bhui

**Affiliations:** 1 Centre for Psychiatry, Wolfson Institute of Preventive Medicine, Barts and the London School of Medicine and Dentistry, Queen Mary University of London, London, UK; 2 Centre for Primary Care and Public Health, Barts and the London School of Medicine and Dentistry, Queen Mary University of London, London, UK; 3 Cathie Marsh Centre for Census and Survey Research, University of Manchester, Manchester, UK

**Keywords:** MENTAL HEALTH, PREVENTIVE MEDICINE, OCCUPATIONAL & INDUSTRIAL MEDICINE, PUBLIC HEALTH

## Abstract

**Objectives:**

To investigate the feasibility of recruitment, adherence and likely effectiveness of an e-learning intervention for managers to improve employees’ well-being and reduce sickness absence.

**Methods:**

The GEM Study (guided e-learning for managers) was a mixed methods pilot cluster randomised trial. Employees were recruited from four mental health services prior to randomising three services to the intervention and one to no-intervention control. Intervention managers received a facilitated e-learning programme on work-related stress. Main outcomes were Warwick Edinburgh Mental Wellbeing Scale (WEMWBS), 12-item GHQ and sickness absence <21 days from human resources. 35 in-depth interviews were undertaken with key informants, managers and employees, and additional observational data collected.

**Results:**

424 of 649 (65%) employees approached consented, of whom 350 provided WEMWBS at baseline and 284 at follow-up; 41 managers out of 49 were recruited from the three intervention clusters and 21 adhered to the intervention. WEMWBS scores fell from 50.4–49.0 in the control (n=59) and 51.0–49.9 in the intervention (n=225), giving an intervention effect of 0.5 (95% CI −3.2 to 4.2). 120/225 intervention employees had a manager who was adherent to the intervention. HR data on sickness absence (n=393) showed no evidence of effect. There were no effects on GHQ score or work characteristics. Online quiz knowledge scores increased across the study in adherent managers. Qualitative data provided a rich picture of the context within which the intervention took place and managers’ and employees’ experiences of it.

**Conclusions:**

A small benefit from the intervention on well-being was explained by the mixed methods approach, implicating a low intervention uptake by managers and suggesting that education alone may be insufficient. A full trial of the guided e-learning intervention and economic evaluation is feasible. Future research should include more active encouragement of manager motivation, reflection and behaviour change.

**Trial Registration number:**

ISRCTN58661009.

**Strengths and limitations of this study**

- Using a mixed methods approach helped us to understand the reasons for why there was a small change in employee well-being related to the intervention.
- There was low adherence to the full intervention among managers.
- Gathering sickness absence and economic data was found to be feasible.
- The interval between the end of the intervention and follow-up of employees was probably too short to allow managers to implement organisational changes likely to lead to changes in employee well-being.
- Considerable organisational change during the study made it a less than ideal context for an intervention to reduce work stress in employees.

## Background

There is empirical evidence including several meta-analyses, showing that the psychosocial work environment in terms of job strain, low social support at work from managers and colleagues, effort-reward imbalance, organisational injustice and job insecurity impacts on employee well-being and risk of sickness absence.^1–4^ There is a consensus that employees’ health is a public health priority and the responsibility of employers and employees as well as health services.^5^ ^6^ So far, evaluations of organisational interventions for workplace stressors are limited. Three reviews of interventions within organisations^7–9^ showed mixed evidence of benefit on health outcomes: van der Klink^10^'s meta-analysis of 48 studies of occupational stress interventions showed that the majority of interventions were delivered to individuals rather than targeting organisations, and often involved cognitive-behavioural techniques.

A review of studies of workplace reorganisation involving increasing skill discretion, team working and decision latitude in diverse occupational groups showed that team working interventions improved the work environment, by increasing support.^11^ At the organisational level, team working interventions have tended to demonstrate improvements in the work environment by increasing support^12^ and some studies of training and organisational approaches to increase participation, decision-making, and work support and communication have reduced sickness absence.^13^

We used an organisational-level intervention based on the Health and Safety Executive (HSE) management standards for work-related stress.^14^ These psychosocial interventions were the first UK approach to reduce the incidence of work-related stress at source by applying a risk assessment process to triggers of work-related stress and found by managers to be a quick and easy method for identifying and resolving problems.^15^ Training managers influences employee well-being; lecture-based educational interventions for supervisors increase supervisor knowledge^16^ and employee well-being.^17^ We anticipated that this intervention might also change manager behaviour. We chose e-learning rather than face-to face-face instruction as an efficient and potentially cost-effective way of training managers. E-learning is supported by studies showing that web-based stress management psychoeducation programmes improve employee job satisfaction,^18^ reduce employee stress compared to controls^19^ ^20^ while computer-presented stress management interventions show similar short-term reductions in stress to instructor-led programmes.^21^

However, there have been insufficient methodologically robust RCTs to test whether organisational-level psychosocial interventions are effective in improving the well-being of employees and reducing sickness absence. Building on the HSE management standards,^14^ this randomised trial of a guided e-learning programme for managers tested the acceptability of the intervention, the feasibility of recruitment, as well as adherence, comprehension and likely effectiveness of the intervention. We used quantitative and qualitative methods. We also tested the feasibility of the collection of sickness absence data and piloted methods of economic evaluation of the intervention in terms of reduced sickness absence and health service costs.

## Methods

### Study design

This study was a pilot cluster randomised trial. The clusters were services belonging to one National Health Service (NHS) Mental Health Trust. Six workplace services were considered for inclusion; two were rejected because of insufficient employment data and dissimilar work. Employees gave informed consent to participate in the study prior to randomisation. After randomisation managers from the intervention clusters were invited to take part in the intervention. A parallel qualitative investigation of key informants, managers and employees was carried out.

### Study population

Participants were employees and managers of an NHS Mental Health Trust. Inclusion criteria were (1) the organisation's ability to provide data on sickness absence and (2) managers allowed internet access at work. Employees who would not remain in the organisation during the study because of long-term sickness, notified pregnancies or fixed-term contracts were excluded.

### Randomisation and blinding

Three workplace services were randomly allocated to the intervention and one to control by an independent statistician. Employees were blinded to whether their managers were in the intervention or control group. It was not possible to blind managers to the study. All study participants received a study information sheet.

### Intervention

The intervention used was the Anderson Peak Performance e-learning package ‘Managing Employee Pressure at Work’, an established e-learning health promotion programme for managers with a focus on the six management standards domains: Change, Control, Demands, Relationship, Role and Support (http://www.andersonpeakperformance.co.uk). This psychosocial programme aims to help managers identify sources of stress, understand the link with mental and physical illness and improve managers’ capacity for helping employees proactively deal with stressful working conditions. The intervention also involved guidance in the form of introductory and follow-up face-to-face sessions from a study facilitator and support by telephone and email. The study facilitator had 2 days training from the e-learning programme developer.

The e-learning programme was designed to help managers understand:

- ▸ The concept of pressure at work, the link with mental and physical ill health, the need to take this seriously and the personal benefits for doing so.
- How to work proactively with their teams to identify collective problems and find solutions.
- How to spot if an employee has a problem and work with the individual to find suitable acceptable solutions.
- How to support individual employees who are experiencing problems.
- Their legal duty of care. How to avoid personal injury claims and how to carry out an HSE compatible risk assessment if required.
- How their own management style may add to or reduce pressure on their employees.

**Figure 1:**
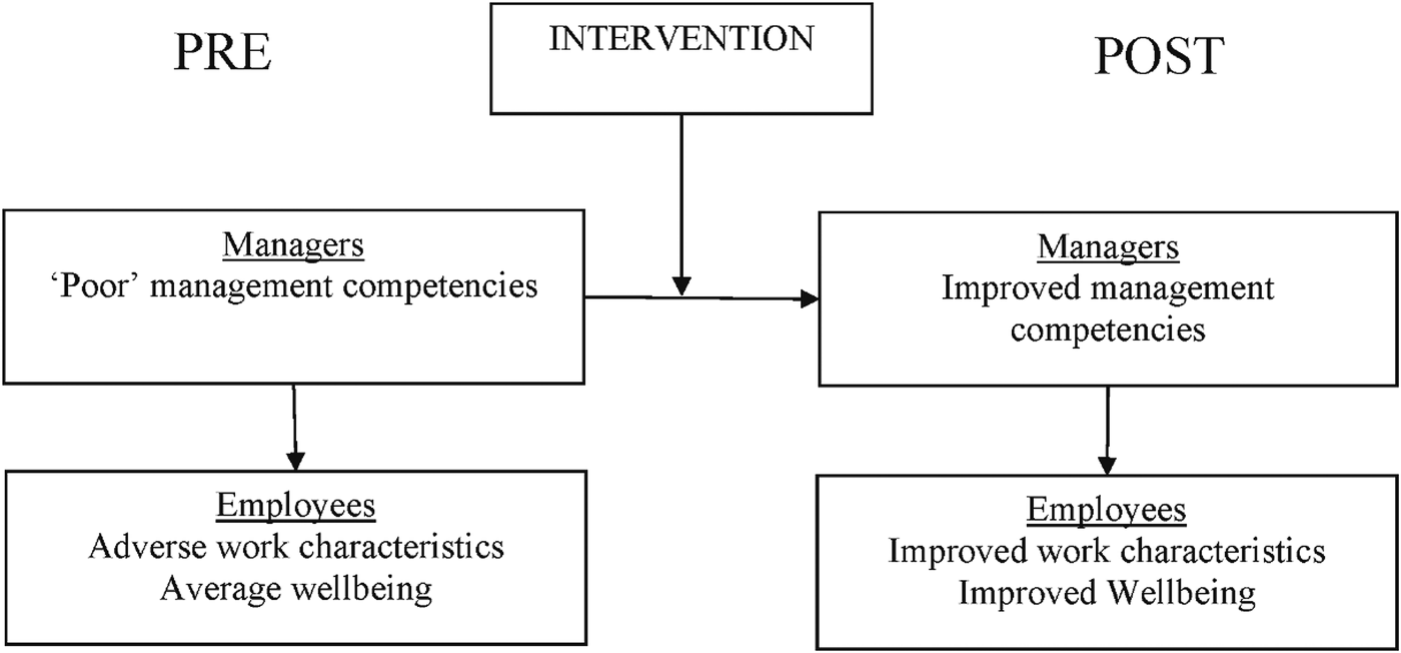
Simplified model of the potential mechanism of effect of the intervention on managers and employees.

The intended mechanism of the intervention was: through participation in the e-learning programme, completion of e-learning activities and consultation with the facilitator, managers would change their behaviour towards employees and workplace conditions. Understanding the stress process, including appraisal and coping, according to the Lazarus model^22^ will provide managers with an understanding of the perceptions and responses to work stressors. The intervention should encourage managers to develop relationship-focused, rather than task-focused, supervisory behaviours and transformational leadership behaviours involving individualised consideration of employees that improve employees well-being and reduce stress.^23–25^ The intervention will help managers reflect on the quality of the supervisor-direct report relationship (Leader-member exchange) which can buffer the negative effect of work stressors on well-being.^26^ Increased well-being would be related to employees taking less sickness absence (see figure 1).

The format of the e-learning programme was a series of linked topics with case examples, additional activities which could be completed outside the e-learning environment. The programme was delivered in weekly to two weekly modules over a 3-month period; the main e-learning content was presented in six separate modules. Managers completed an online quiz before and after the programme. Managers received a certificate for successful completion of the programme as part of their continuing professional development.

### Control cluster

The managers in the control cluster received no intervention.

### Data collection

Employees were recruited between June and October 2013 by the local research team and were asked to complete a baseline questionnaire. The participants who responded were asked to complete the follow-up questionnaire 3 months later, between January and April 2014. Participants were invited by email to login to the questionnaire online. In case of non-response, two automated email reminders were sent 7 days apart, followed by one personalised email reminder, then if no response was received, local research staff attempted phone contact with the participant and paper questionnaires were offered to non-responding employees.

Anonymised data on sickness absence was collected directly from human resources, and covered absences during the periods of 1 May and 31 July 2013 for the baseline assessment and between 2 January 2014 and 30 April 2014 for the follow-up. Uptake data on managers’ adherence to the intervention, and their e-learning quiz scores, were logged by the system and reported by the organisation hosting the programme.

### Qualitative data collection

Fourteen in-depth interviews were carried out with key informants from the Trust, the steering committee and people with expertise in work related stress. In-depth interviews were carried out between February and April 2014 with a sample of managers. A purposive approach was adopted to ensure a heterogeneous sample, including men and women from the intervention and control group clusters. Twenty-one of the 41 managers in the intervention clusters who had consented to participate in the study were approached for interview and 11 agreed; a response rate of 52% among intervention group managers. Eight managers from the control cluster were invited to interview and two agreed. Thus a total of 13 in-depth interviews were undertaken with managers, 10 women and 3 men (reflecting the female/male ratio in the managers participating in the trial). The interviews were audio recorded and transcribed. Interviewed managers were also invited to a ‘feedback’ meeting at the end of the data collection; this meeting was convened as a focus group discussion, and was audio recorded and transcribed. Three managers attended.

A similar purposive approach to sampling was adopted for selecting employees for interview. To avoid possible influence on employees’ questionnaire responses, we approached employees for interview after they had completed their follow-up questionnaires. Thirty-six employees from across the four clusters in the study were invited for interview (from the sample of 163 employees who had completed their follow-up questionnaires by this time). Ten employees responded (a relatively low response rate of 28%), but we were unable to arrange interviews with two of these employees and subsequently undertook a total of eight employee interviews (six women, two men, again reflecting the male female ratio in the trial employee sample). The sample comprised two employees from each of the four clusters in the study.

We adopted a narrative approach to interviewing, meaning that interview questions encouraged respondents to recount stories of specific, anonymised cases and incidents, as a way of eliciting a rich and reflective account of the complexities of managing stress at work. A narrative methodology focuses on concrete practice rather than, as is often the case with other interview methods, on abstract perspectives.^27^

Observational data was collected from nine meetings of managers during the study (one preliminary meeting at which the study was introduced, six facilitator-led support meetings and two dissemination meetings at the end of the study), and from the project steering committee and team meetings. Additional data from employees was collected from a ‘free text’ box in the baseline and follow-up questionnaires.

Data analysis took place concurrently with data collection. The two qualitative researchers engaged in close readings of the transcripts of interviews and meetings, observational field notes and associated documentation. We individually and collectively identified themes emerging from the data, both within subsets of our data (ie, themes emerging from key informant interviews, from manager interviews and from employee interviews) and across the data set. We discussed our preliminary findings with members of the study team, individually and at team meetings and with the steering committee, and drew on these discussions to interrogate our data further and develop our in-depth analysis.

We adopted established principles for assuring validity of qualitative research: ‘trustworthiness’ (through transparency about processes of data collection and analysis), ‘thick description’ (through collecting in-depth narrative accounts), ‘reflexivity’ (through ongoing discussion about emerging findings and interpretation) and ‘multidimensionality’ (through drawing on data from a variety of sources).^28^

### Outcome measures

Two primary outcome measures were assessed.

- Employee well-being: pre–post changes in levels of well-being were assessed by the Warwick Edinburgh Mental Wellbeing Scale (WEMWBS),^29^ a 14-item scale assessing positive mental health.
- Sickness absence: (primary cluster-level outcome) pre–post changes in sickness absence were monitored using the existing reporting system of the NHS Trust and local Social Services. Sickness absence was measured in days excluding absences greater than 21 days.

The secondary outcomes assessed included:

- Self-report sickness absence: short-term (<7 days) and medium-term (7–21 days) sickness absence.
- Psychological distress measured by the 12-item General Health Questionnaire (GHQ12).^30^ In the baseline questionnaire the wrong item responses were reproduced for Question 12 (happiness) on the baseline GHQ where “Not at all/No more than usual/Rather more than usual/Much more than usual” was presented to the employees instead of “More so than usual/About same as usual/ Less so than usual/Much less than usual”. For the analysis the first 11 items were used and multiplied by 12/11 for both baseline and follow-up.
- Self-reported psychosocial work characteristics were assessed using standardised assessment tools: (1) Karasek's Job Content Questionnaire, measuring job strain (decision latitude and psychological demands), work social support^31^ and (2) effort-reward imbalance^32^ using abbreviated versions developed for use in birth cohort studies.^33^
- Knowledge gained by managers from the programme assessed by a quiz embedded in the e-learning.

### Statistical analyses

The analysis was carried out using Stata V.12. These analyses were mainly descriptive; no formal statistical analyses were conducted to compare the effectiveness of the intervention, although CIs are presented. Participation rates are presented overall, and 95% CIs for rates have been presented without adjustment for clustering. Effectiveness comparing intervention and control clusters was estimated using a random effects model with restricted maximum likelihood estimation. As the random effects model assumes a large sample for the number of clusters, the CIs were calculated using the SE from the model and the t-distribution with 2 df instead of the Normal distribution. Post hoc analyses were carried out to assess changes in well-being scores for employees of managers who did or did not engage with the intervention, as well as for employees of managers who changed position during the course of the study. This analysis used a random effect model but did not adjust for the small number of clusters as the comparison was within rather than between clusters.

The study aimed to recruit 120 individuals from 4 clusters and anticipated that 100 individuals would consent. This would allow the response rate to be estimated to within 3.8 percentage points, for example, 76.1–83.9%. Measurements of intervention acceptability were estimated from those individuals who consented and are randomised to the intervention anticipated to be 300 individuals. If the take-up is 80% this would be estimated to within 4.5% points. We envisaged recruiting 30–40 managers, each responsible for 5–20 employees. As one of our aims was to understand the intervention processes we allocated more clusters to intervention than control.

### Costs

A microcosting of the guided e-learning programme for managers included a construction of the costs associated with setting up and delivering the programme. The course running costs included facilitator's wages and travel expenses, managers’ salaries and travel expenses, administration costs (administrator's and meeting organisers’ salaries), software licence fee and telephone/internet bills. The cost of the intervention also included the cost of training the facilitator.

## Results

**Figure 2:**
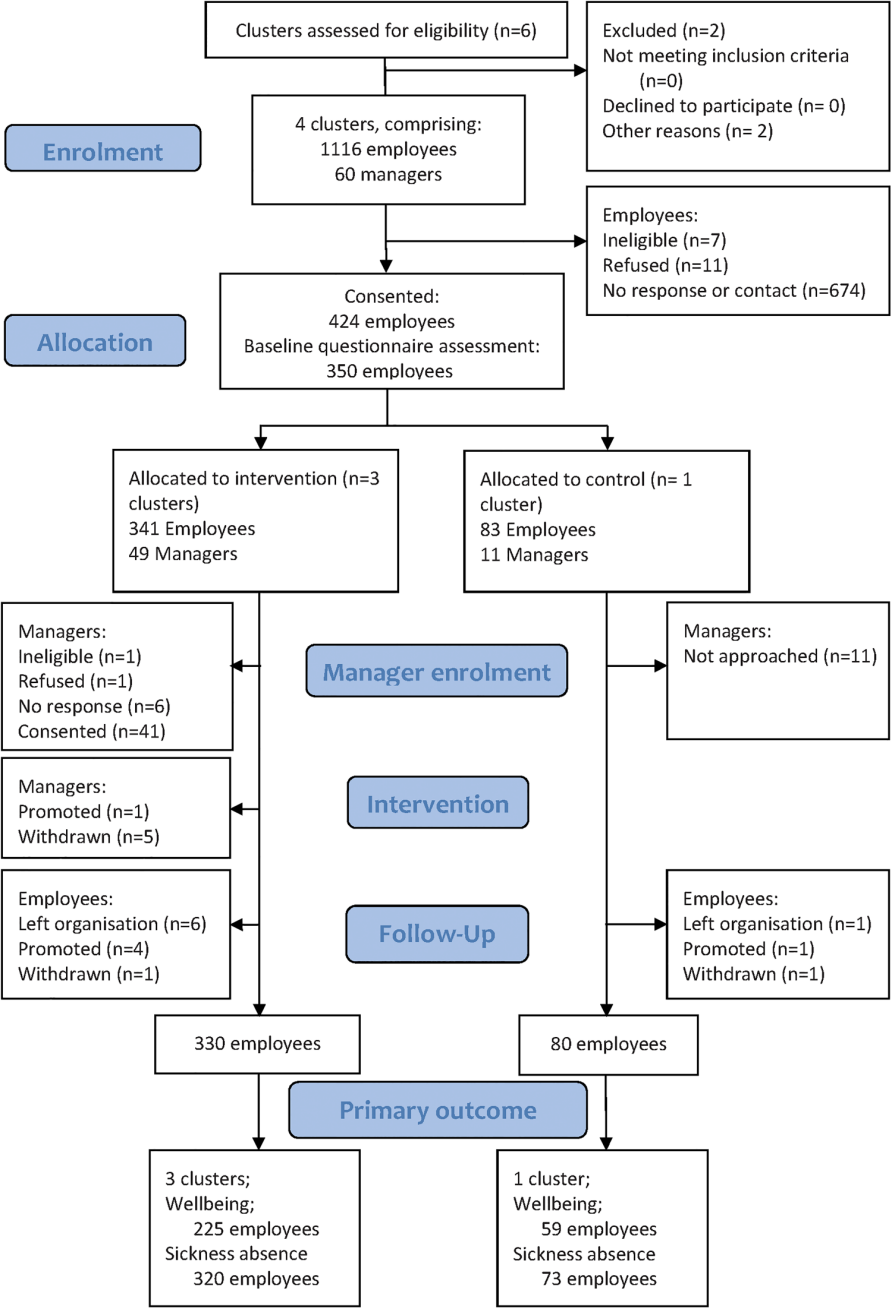
Participant flow diagram.

Figure 2 presents the flow diagram of participants during this trial. Employees were recruited by the local research team, who visited the various teams across the four clusters and attended local team meetings to introduce the study. As teams were spread over a large geographical area, many employees were working off-site, and the meetings were never attended by all staff, not all employees could be contacted personally. The local research team reported contacting 649 employees during these visits of whom 424 (65%, 95% CI 62% to 69%) employees consented. Consent rates for individual cluster varied from 56% to 72% both extremes being intervention clusters. Baseline questionnaires were completed for 350 (83%, 95% CI 79 to 86%). The fall-off in response may be because employees changed their minds about participation or were put off by the on-line questionnaire. A total of 277 were completed online and 69 completed paper only and four started online and completed on paper. At follow-up 291 participants (69%, 95% CI 64% to 73%) completed the questionnaire of whom 284 had completed the WEMWBS, the primary end point. Follow-up rates per cluster varied from 59% to 77%.

Baseline and follow-up sickness absence was available for 393 employees (93%, 95% CI 90% to 95%); 368 employees from the participating Trust HR database and 25 employees from three local council HR departments. Reasons for non-availability of sickness absence data were: participant withdrawal, staff on contracts with no centralised sickness absence records or administrative reasons.

Baseline population demographic characteristics were comparable to the Trust-wide demographic profile. Percentages for trust staff versus GEM participants were 79% versus 76% female; 40% versus 38% part-time employees; 35% versus 27% over 50 years of age.

The demographic characteristics of employees were broadly similar between the intervention and the control clusters (table 1) although there were a few minor differences. A greater proportion of women were in the control cluster (85% vs 74%) and fewer employees aged 50 or more (31% vs 40%) and a lower proportion felt that their job interfered with their family life (45% vs 58%). Finally, more employees in the control cluster were band 7 or higher (25%) than in the intervention cluster (15%).

**Table 1:** Demographic, health and lifestyle characteristics of employees at baseline

	Control n=67 1 cluster	Intervention n=283 3 clusters	Total n=350 % (range of % or mean within each cluster)
Female	57 (85)	209 (74)	76 (60 to 85)
Age			
Employees aged over 50	21 (31)	112 (40)	38 (31 to 47)
Employees under 30	6 (9)	27 (7)	–
Employees 30 to 39	9 (13)	52 (18)	–
Employees 40 to 49	31 (46)	98 (35)	–
Employees 50 to 59	21 (31)	102 (36)	–
Employees who are married or cohabiting^1^	49 (73)	210 (74)	74 (68 to 79)
Employees with children	32 (48)	117 (41)	43 (37 to 48)
Employees who support a family member^2^	14 (21)	60 (21)	21 (16 to 28)
Employees who report family life interfering with work^2^	17 (26)	80 (28)	28 (26 to 30)
Employees who report job interfering with family life*^,3^	30 (45)	160 (58)	54 (45 to 68)
Part time employees*^,2^	17 (26)	78 (28)	27 (24 to 32)
Number of hours worked per week (mean, SD)^4^	35.3 (8.7)	36.6 (6.8)	36.4 (35.3 to 36.9)
Employee band 7 or higher salary^5^	17 (25)	42 (15)	17 (8 to 25)
Employees who are in charge of others^6^	12 (18)	48 (17)	17 (16 to 18)
Employees with poor health†	11 (16)	44 (16)	16 (10 to 21)
Employees with disability or limited activities	10 (15)	39 (14)	14 (11 to 17)
Employees who smoke^8^	11 (16)	60 (21)	20 (16 to 30)
Employees reporting problems with drinking^9^	8 (12)	32 (12)	11 (7 to 15)
Self-reported absence in the past 3 months‡	14 (22)	74 (26)	25 (21 to 31)
Number of days of absence reported (mean, range)^10^	2.7 (0 to 90)	1.9 (0 to 70)	2.0 (1.1 to 2.7)
Well-being score (mean, SD)	50.4 (8.0)	51.0 (8.3)	50.8 (49.5 to 51.7)
GHQ12 score (mean, SD)§	3.0 (3.3)	2.8 (3.4)	2.8 (2.5 to 3.0)
Employees who have GHQ12>3‡	26 (39)	98 (35)	35 (31 to 41)
Perceived social support :some lack¶	13 (19)	61 (22)	21 (18 to 25)

Missing data; Control/Intervention 1=0/1; 2=1/3; 3=1/5; 4=0/9; 5=0/3; 6=3/2; 7=1/5.

Missing data Control/intervention 8=0/3; 9=0/7;10=0/3 plus 4/6 preferred not to answer question; 11=5/14; 12=0/4.

Coding.

*Defined as proportion of employees who reported interference as ‘to some extent’, or ‘a great deal’.

†Defined as proportion of employees who self-reported general health as ‘fair’, ‘bad’ or ‘very bad’.

‡Expressed as the number of days absence for the entire group.

§Based on 11 items.

¶One or more questions out of 4 not ‘Certainly true’.

GHQ, General Health Questionnaire.

Health at baseline was similar in employees from both intervention and control clusters (table 1). Notably, both clusters had a large proportion of employees who scored above the accepted caseness threshold on the GHQ, 39% in the control and 35% in the intervention cluster.

Psychosocial work characteristics at baseline, including supervisor relationships and information, job insecurity, work social support and job strain were similar between the intervention and control clusters and there were few differences between psychosocial characteristics in the intervention and control clusters at follow-up.

There was little difference in the baseline characteristics of employees between those who completed and did not complete the follow-up questionnaire (table 2). Female employees were slightly more likely to have completed the questionnaire (77% vs 73%), as were employees aged over 50 (39% vs 34%), working part-time (28% vs 22%) and employees in job band 7 or above (19% vs 7%, p<0.025). Completers were less likely to smoke (32% vs 18% p<0.014).

**Table 2:** Demographic and well-being characteristics and outcomes for employees who completed and did not complete the follow-up questionnaire

	Completed n=291 (%)	Not completed n=59 (%)	p Value
Female	223 (77)	43 (73)	0.54
Aged over 50 years	113 (39)	20 (34)	0.48
Part-time employees	82 (28)	13 (22)	0.35
Job band 7 or above	56 (19)	4 (7)	0.025
Employees with self-reported ill health	47 (16)	8 (14)	0.62
Employees with disability or limited activities	44 (15)	5 (8)	0.18
Employees who smoke	52 (18)	19 (32)	0.014
Employees reporting problems with drinking	36 (13)	4 (7)	0.25
Self-reported sickness absence in past 3 months	73 (26)	14 (25)	0.89
Well-being score at baseline (mean, SD)	50.8 (8.3)	50.8 (7.1)	0.96
GHQ12 score at baseline based on 11 items (mean, SD)	2.9 (3.5)	2.4 (3.0)	0.29
Proportion employees with GHQ12 score >3	106 (37)	18 (31)	0.33

GHQ, General Health Questionnaire.

Wellbeing score on the WEMWBS declined from 50.4 to 49.0 in the control and from 51.0 to 49.9 in the intervention clusters. The overall intervention effect after adjusting for clustering and baseline value was small with a difference of 0.5 points between the intervention and control cluster (95% CI −3.2 to 4.2; table 3). There was no evidence of any beneficial effect of the intervention on GHQ score, supervisor relationships, or supervisor support (table 3). We examined the intervention effect in subgroups of employees: employment grade five or less compared to six and above, full-time versus part-time employees and whether they had worked for the organisation for less or equal to 2 years or 3 years or more. However, we were too underpowered to derive reliable estimates and the CIs were wide. Intracluster correlations coefficients estimated using the models for the analysis in table 3 were WEMWBS 0.0000; Days off sick HR data 0.0003; Days off sick self-report 0.008; GHQ 0.012; Supervisor relationship 0.0000; supervisor support 0.008. In all cases the CIs were very wide indicating very unreliable estimates.

**Table 3:** Comparison of primary and secondary outcomes in intervention and control clusters

	n	Baseline	Follow-up	Difference between baseline and follow-up (95% CI)	Intervention effect adjusted for baseline and clustering*
		Mean (SD)	Mean (SD)		
*Primary outcomes*					
Well-being score					
Control	59	50.4 (8.0)	49.0 (8.5)	−1.4 (−2.8 to 0.0)	
Intervention	225	51.0 (8.3)	49.9 (8.3)	−1.1 (−1.9 to 0.2)	*0.5 (*−*3.2 to 4.2)*
Days off sick from HR data†					
Control	66	0.9 (2.0)	1.0 (1.7)	0.1 (−0.4 to 0.6)	
Intervention	294	1.2 (3.2)	1.6 (3.7)	0.4 (−0.1 to 0.9)	*0.6 (*−*1.4 to 2.6)*
Days off sick self-report†					
Control	51	1.2 (3.5)	1.3 (3.8)	0.1 (−0.8 to 0.9)	
Intervention	198	1.0 (3.0)	1.3 (3.4)	0.3 (−0.3 to 0.9)	*0.1 (*−*2.2 to 2.4)*
*Secondary outcomes*					
GHQ					
Control	59	3.2 (3.4)	2.9 (3.7)	−0.3 (−1.1 to 0.4)	
Intervention	216	2.8 (3.5)	2.9 (3.5)	0.0 (−0.4 to 0.5)	*0.2 (*−*2.0 to 2.5)*
Supervisor relationships					
Control	59	74 (21)	75 (19)	0.9 (−5.6 to 7.4)	
Intervention	224	72 (20)	71 (21)	−1.5 (−3.8 to 0.9)	−3.3 (−14.1 to 7.5)
Supervisor support					
Control	59	87 (23)	86 (21)	−0.8 (−8.7 to 7.0)	
Intervention	228	80 (23)	80 (24)	−0.1 (−3.1 to 2.8)	−3.2 (−19.2 to 12.9)

*The difference in well-being score and mean days off sick between intervention and control arms, adjusted for baseline and clustering.

†Excluding those off sick for more than 21 days at baseline.

GHQ, General Health Questionnaire.

Of 41 managers, only 21 (51%) achieved the minimum requirements of having completed three of the six main e-learning modules in order to qualify as ‘adherent’. The relatively low adherence of managers also meant that only 120 out of 225 intervention group employees who provided complete WEMWBS data for analysis had a manager who adhered to the intervention. Employees whose managers were not adherent to the intervention, either through not consenting or not completing at least three modules, had worse WEMWBS scores at baseline than adherent managers (49.8 vs 52.0). During the study period, a fall in the WEMWBS scores was seen for employees in both arms but it was a significantly smaller reduction among managers who engaged with the intervention (−0.7 vs −1.6 with an adjusted difference of 1.6, 95% CI 0.1 to 3.2). There was a small decline in mean GHQ scores between baseline and follow-up among employees whose managers were adherent compared to employees of non-adherent managers (−0.2 vs 0.3 with an adjusted difference of −0.7, 95% CI−1.5 to −0.0).

Mean days off sick at baseline were 1.2 in the intervention cluster and 0.9 in the control cluster rising to 1.6 and 1.06 respectively (table 3). Self-report of days off sick were 1.2 in the control cluster at baseline and 1.0 in the intervention cluster (table 3). At follow-up self-reported days off sick had increased to 1.3 in the control and 1.3 in the intervention cluster. An intervention effect of 0.1 was observed (95% CI −2.2 to 2.4).

No harms or major adverse effects were reported during the study, neither to the facilitator, the qualitative researcher or another member of the research team. There were no reported adverse effects of either the trial or the intervention.

The itemised intervention costs are listed in online supplementary tables. The total cost of the intervention was £20 963.

Two estimates of intervention costs were used in the economic analyses: one based on the number of managers randomised to the intervention group (n=49) and another based on the lowest number of managers (n=18) who participated in any one of the three parts of the course. The average cost per participant (manager and employee) estimated with and without facilitator training is shown in online supplementary tables S1 and S2. The cost of the intervention per employee varied between £81 and £153 depending on the assumptions made.

The qualitative study identified the following sources of workplace stress among managers and employees: organisational change and organisational culture, job insecurity, poor communication, insufficient resources to deal with the volume of work, the physical environment, the nature of mental health work and the pressures of family life events and ill-health.

The qualitative study found that overall the intervention and trial were acceptable to managers and employees who took part in the study. The e-learning programme was considered easy to access, straightforward to use and the content relevant. Managers were ambivalent about e-learning, identifying both benefits and disadvantages of its flexibility. They favoured a ‘blended’ approach, seeing e-learning as a supplement to, rather than a replacement for, other learning methods and welcomed the opportunity to share experiences and for peer learning in the face-to-face group meetings. The key identified value of the e-learning programme was that it ‘backed up’ existing knowledge and encouraged reflection on managerial practice rather than imparted new knowledge. When asked about the skills that managers need to deal with stress and in recounting specific instances of workplace stress, managers and employees focused on the value of experiential knowledge, on the need to ‘juggle’ competing demands and roles and on affective qualities such as trust, empathy, compassion and approachability (‘emotional intelligence and sensitivity’) rather than the management competencies explicitly covered in the e-learning modules (see box 1).

Box 1 **A manager's story of helping an employee manage stress**

It's more of a personal nature for this member of staff. She's going through a very difficult break up of a marriage, got young children too…it's all blown up and all…really struggling, really having difficulties with it. I'm going out to see her on a fairly regular basis—I've been out to see her today, actually. Going out, giving all the support I can refer her to occupational health, refer her to staff support. It's a really difficult one because I'm sitting there saying, ‘Yes, yes. I hear that you're not ready to come back. Yes, I hear what you're saying to me,’ but on the other side of that is the fact that there's a service need. She had a caseload of patients that we've had to share out with other people now, not everybody wants to go to another therapist. Therapy's quite individualised and quite thought provoking, and you're sharing your soul to the devil, so to speak, aren't you? That's how it feels. So that's difficult because it's that balance of I hear what you're saying, you're in a really horrible place, I can't imagine anything worse for you, but on the other side of that, I've got to get you back into work somehow…I think I've had to draw on compassion. I think I've had to draw on knowing the policy, knowing what I can and cannot allow her to do. The return to work policy, the phased return, all of that, I've had to look on that. I think I've had to draw my own personal beliefs and my own personal values, really, and be able to stand up and say, ‘I hear what you're asking as a Trust. I hear what you're saying as a Trust but I'm the person that's in there, I'm the person that's dealing with this individual, you know, I'll bring her in to fail and that's how I feel at the moment. I think she's too fragile, too vulnerable to come back in at this precise moment but I'm also aware that if I take that to a more senior manager they may say I hear what you're saying but she needs to get back in. (M6)

Managers reported insufficient time to engage with the intervention and a lack of senior management ‘buy-in’. Some skepticism was expressed about the extent to which a brief guided e-learning intervention could be expected to impact on long-standing attitudes and beliefs about stress in the workplace. The intervention was thought to need better integration into organisational processes and practice.

## Discussion

We carried out a pilot study of a guided e-learning intervention for managers designed to improve employee well-being and reduce levels of sickness absence in a mental health trust. We recruited sufficient employees and the managers who took part found the guided e-learning intervention and the trial acceptable. We piloted methods for collecting sickness absence data and data for economic evaluation and found this was feasible. There was only a very small effect of the intervention on employee well-being and little effect on sickness absence but the study was not powered to examine this definitively.

### Interpretation of findings

The small difference in well-being between the intervention and control groups may be due to several factors: (1) insufficient interval between intervention and follow-up employee well-being measurements, (2) choice of well-being measure, (3) the confounding effects of considerable organisational change taking place during the study, including a number of managers being reallocated during the study, (4) a poorer than expected intervention uptake rate among managers and (5) shortcomings in the intervention itself and the underpinning theory of change. It is possible that the intervention had some impact on *manager* well-being, but this was not measured in the study. There is little comparative data on how programmes like this might affect well-being and work. An RCT of a participatory intervention involving action planning with nurses, sharing good practice and obstacles, was associated with changes in work characteristics but not mental health^34^ and a participatory risk management intervention in an Australian public sector organisation was associated with significant improvements in job design, training and morale and a reduction in sickness absence duration.^35^

The finding that employees in the intervention cluster whose managers completed the intervention had higher well-being scores, both at baseline and follow-up implies that the more engaged managers already had employees with higher levels of well-being and that it may be that employees relating to the less engaged managers might have shown greater increase in well-being related to the intervention. The overall decline in well-being across the study in both groups may reflect the wider changes in the NHS of continuing reorganisation, recession, declining resources and job insecurity.

In view of these results, and our review of educational and workplace stress literature, we consider that our original theory of change, that a largely instructional educational intervention would lead to behaviour change in managers and increased well-being in employees, requires refinement. The inclusion of affective engagement and motivational elements in the programme may be as important as the knowledge imparted about good management practice.

It is possible that our study does not represent a full and fair test of the intervention. Qualitative investigation revealed that the managers who engaged in the study were atypical of managers generally, being highly experienced, in post for long periods of time and conversant with psychological stress in the workplace. Thus it may not be realistic to expect change in managers who are already functioning at a high level and found the intervention too basic. By contrast, if we had been able to engage the managers who did not adhere and included new inexperienced managers from organisations unfamiliar with workplace stress we might have shown different findings.

Health economics data collection was shown to be feasible, but will require a full trial for a detailed cost-benefit analysis, a recent review shows mixed results of the cost-effectiveness and financial return of worksite mental health interventions.^36^

### Strengths and limitations

Strengths of the trial included good recruitment and retention, complete questionnaire and sickness absence data and complementary qualitative and economic analyses. There were some limitations of the study that worked against the trial showing an effect. The setting for the trial—an NHS Trust went through a major reorganisation during the course of the study. This may have impacted on the acceptance of the study by employees and managers and potentially put off managers from fully engaging with the intervention. This also meant that some managers were reallocated during the study, interfering with recruitment and retention of managers. A limitation of the qualitative sample was that it only included one ‘non-adherent’ manager. Furthermore, as consent had not been gained for this purpose, we were unable to approach managers in the Trust who did not participate in the study, although their views would have been of interest.

Although we had initial meetings with senior managers to embed the study in the local work culture we could have had more buy-in from senior managers to enable them to permit their middle managers to spend more time on the intervention. We had a poorer than expected intervention uptake rate among managers which may partly relate to the work pressures cited above but may also be that we failed to attract the less committed managers who might have benefited more from the intervention. The interval between intervention and follow-up employee well-being measurement may have been too short thus not allowing sufficient time for the managers to implement changes in management style they learnt from the intervention. It is also possible that we were limited by shortcomings in the intervention itself and the underpinning theory of change. The e-learning programme may require some more active elements that encourage behaviour change in managers. On the other hand, the subgroup analysis suggested that the intervention appeared to work among employees whose managers adhered to the intervention.

### Implications for practice and research

The incorporation of qualitative investigation in this project with quantitative analysis has allowed us to explore the recipients’ response to the intervention, explore the reasons for the trial findings and question the appropriateness of the underlying theory (see Russell J, Berney L, Stansfeld S, *et al.* The role of qualitative research in adding value to a randomised control trial: lessons from a pilot study of a guided e-learning intervention for managers to improve employee well-being and reduce sickness absence. Paper to be submitted to BMC Health Services Research). It is too early for clear implications for practice but in further research we would place a greater emphasis on affective engagement and experiential learning for managers. Thus we propose that this type of education alone is insufficient to change managers’ behaviour and that we need a more active approach. In fact the managers did not fully engage with all the learning activities in the e-learning programme and did not all attend the two face-to-face meetings—and these were not included as criteria for adherence. There is scope to include elements that affectively engage the manager with the programme, encourage manager reflection and behaviour change. This could include managers undertaking self-assessment of their skills and sharing these assessments with their peers to encourage behaviour change.^37^ Greater engagement with senior managers might help to embed the intervention in the local work culture, increase acceptance of use of the intervention by managers, including those who are reluctant to take part and giving them permission to spend more time on the intervention. Increasing the interval between intervention and follow-up data collection, to allow more time for the intervention to take effect, would make the study design more robust. A future study could be part of a comprehensive approach that attempts to reduce work-related risk factors, promote mental health emphasising the positive aspects of work and address existing mental health problems.^38^
